# Analysis of risk factors and the predictive value of a nomogram model for coronary heart disease in patients with rheumatoid arthritis

**DOI:** 10.3389/fcvm.2025.1558012

**Published:** 2025-06-09

**Authors:** Guozhu Che, Xing Zhao, Haizhuan An, Yanyan Wang, Qianyu Guo, Ke Xu

**Affiliations:** Department of Cardiovascular Medicine, Shanxi Bethune Hospital, Shanxi Academy of Medical Sciences, Tongji Shanxi Hospital, Third Hospital of Shanxi Medical University, Taiyuan, Shanxi, China

**Keywords:** rheumatoid arthritis, coronary heart disease, nomogram model, risk factors, predictive analysis

## Abstract

**Background:**

Rheumatoid arthritis (RA) is associated with an elevated risk of coronary heart disease (CHD) due to a complex interplay of traditional cardiovascular risk factors and RA-specific mechanisms. This study aimed to identify key risk factors for CHD in RA patients and develop a nomogram model for individualized risk prediction.

**Methods:**

A retrospective study was conducted involving 258 RA patients, including 32 with CHD and 226 without CHD, admitted between January 2021 and August 2024. Demographic, clinical, and laboratory data were collected. Multivariate logistic regression analysis identified independent risk factors, which were incorporated into a nomogram model. The model's performance was evaluated using the receiver operating characteristic (ROC) curve, calibration plots, and decision curve analysis (DCA). Internal validation was performed using bootstrap resampling.

**Results:**

Key risk factors for CHD in RA patients included hypertension, HbA1c, RA duration, carotid plaque burden, uric acid, and ECG abnormalities. The nomogram demonstrated excellent discriminative ability, with an area under the ROC curve (AUC) of 0.868 (95% CI: 0.819–0.916) and robust calibration (*P* = 0.908). Internal validation confirmed its reliability (AUC = 0.866). DCA indicated that the nomogram provided superior clinical utility by optimizing the net benefit across a range of threshold probabilities.

**Conclusions:**

This study identified hypertension, elevated HbA1c, prolonged RA duration, carotid plaque burden, increased uric acid levels, and ECG abnormalities as significant risk factors for CHD in RA patients. A nomogram prediction model incorporating these factors was developed, exhibiting outstanding discriminatory and calibration capabilities.

## 1 Introduction

Rheumatoid arthritis (RA) is a chronic, systemic autoimmune disease characterized by persistent inflammation of the synovial joints, which often leads to joint deformities and significant disability. Beyond musculoskeletal manifestations, RA is increasingly recognized as a condition with profound systemic implications, particularly in its association with cardiovascular diseases (CVDs) (1, 2). Among these, coronary heart disease (CHD) is a leading cause of morbidity and mortality in patients with RA. The inflammatory milieu and immune dysregulation inherent to RA not only drive joint damage but also contribute to the accelerated development of atherosclerosis and coronary artery disease, positioning RA patients at a substantially elevated risk of CHD compared to the general population. Coronary heart disease encompasses a spectrum of conditions caused by impaired blood flow to the myocardium, typically due to atherosclerotic plaque formation in the coronary arteries (3, 4). In RA patients, the risk of CHD is amplified by both traditional cardiovascular risk factors—such as hypertension, hyperlipidemia, and smoking—and RA-specific factors, including chronic systemic inflammation, autoantibodies, and long-term corticosteroid use. Proinflammatory cytokines, such as tumor necrosis factor-alpha (TNF-α) and interleukin-6 (IL-6), play a pivotal role in the progression of both RA and atherosclerosis, creating a shared pathological pathway that exacerbates cardiovascular risks in these patients. Despite advancements in RA management, including the use of biologic disease-modifying antirheumatic drugs (bDMARDs), the burden of CHD remains disproportionately high in this population (5–7). Although bDMARDs can attenuate systemic inflammation, their long-term impact on cardiovascular outcomes remains heterogeneous, possibly due to variable effects on immune-mediated vascular injury (8). These RA-specific factors may contribute to residual cardiovascular risk even in patients receiving adequate anti-rheumatic therapy (9, 10).

The identification and analysis of risk factors are critical for the early detection and prevention of CHD in RA patients. Traditional cardiovascular risk assessment tools, such as the Framingham Risk Score, often underestimate the cardiovascular risk in RA patients because they fail to account for the additional impact of RA-specific factors. This underscores the need for more tailored risk prediction models that integrate both general and disease-specific factors to provide a comprehensive assessment of CHD risk in RA patients. Nomogram models have emerged as valuable tools for individualized risk prediction in various clinical settings (11, 12). By incorporating multiple predictors into a user-friendly graphical interface, nomograms provide personalized risk estimates that can guide clinical decision-making. In the context of RA-associated CHD, nomogram models offer the potential to integrate traditional cardiovascular risk factors, RA-specific variables, and systemic inflammatory markers into a single predictive framework. Such models could improve risk stratification and inform targeted interventions aimed at reducing cardiovascular events in RA patients (13, 14).

This study aims to analyze the risk factors associated with CHD in RA patients and to evaluate the predictive value of a nomogram model specifically designed for this high-risk population. By addressing the limitations of conventional risk assessment methods, this research seeks to provide a more accurate and comprehensive approach to predicting CHD risk in RA patients. The findings have the potential to enhance early detection and prevention strategies, ultimately improving cardiovascular outcomes in this vulnerable group.

## 2 Methods

### 2.1 Study design

This retrospective study was conducted at our hospital to analyze the risk factors and evaluate the predictive value of a nomogram model for CHD in patients with RA. The study included patients admitted between January 2021 and August 2024. Participants aged 18 years or older with complete and reliable medical records, including demographic data, RA disease characteristics, CHD-related parameters, and laboratory results, were included. Written informed consent was obtained from all participants or their legal representatives prior to their inclusion in the study. Exclusion criteria were applied to eliminate confounding factors. Patients with other autoimmune diseases, such as systemic lupus erythematosus (SLE) or systemic sclerosis (SSc), were excluded. Additionally, individuals with a history of severe comorbidities, including end-stage renal disease, advanced malignancies, or other life-threatening conditions, as well as those with active infections or acute inflammatory conditions unrelated to RA at the time of enrollment, were excluded. A total of 258 RA patients were included in the study, comprising an observation group of 32 patients diagnosed with CHD and a control group of 226 patients without CHD. The study design, intent, and protocols adhered to the STROBE (Strengthening the Reporting of Observational Studies in Epidemiology) guidelines (15). Informed consent was obtained from all subjects and/or their legal guardian(s). This study underwent thorough ethical review and approval by the ethics committees of Shanxi Bethune Hospital, Shanxi Academy of Medical Sciences, Tongji Shanxi Hospital, and the Third Hospital of Shanxi Medical University. Conducted in strict accordance with the Declaration of Helsinki and relevant guidelines, all study methods, design, performance, and reporting maintained the highest ethical standards. Measures were taken to ensure data confidentiality and the protection of participant privacy by removing all personal identifiers.

### 2.2 Diagnostic criteria for rheumatoid arthritis and coronary heart disease in study participants

The diagnosis of RA was established according to the 2010 American College of Rheumatology/European League Against Rheumatism (ACR/EULAR) classification criteria, ensuring the inclusion of patients meeting internationally recognized standards. CHD was confirmed through a comprehensive diagnostic approach that included clinical symptom assessment, electrocardiography, echocardiography, myocardial enzyme profile evaluation, and imaging studies. A definitive diagnosis of CHD required evidence of coronary artery stenosis of ≥50%, as determined by coronary angiography or coronary computed tomography angiography (CTCA).

### 2.3 Assessment of carotid intima-media thickness and plaque scoring

Carotid intima-media thickness (IMT) and plaque burden were assessed using the carotid plaque crouse score. Measurements were performed with the patient in a supine resting position, utilizing a 9l linear-array transducer to examine the carotid arteries. The evaluation included six regions: the left and right common carotid arteries, carotid bulbs, and internal carotid arteries. For IMT analysis, the maximum value among these six regions was selected. For IMT (16), the maximum value across all segments was recorded. A focal thickening with a maximal IMT ≥1.5 mm was defined as a plaque, consistent with conventional ultrasonographic criteria.

For plaque scoring (17, 18), the maximum thickness of each discrete plaque at each segment was measured. The sum of the maximum thicknesses of all detected plaques was calculated to yield the Crouse score, representing the total plaque burden. When plaques were present in the same anatomical segment on both sides, their maximum thickness values were summed bilaterally for that segment. This method provides a reliable estimation of cumulative atherosclerotic load and has demonstrated predictive value for cardiovascular events in both general and high-risk populations.

### 2.4 Data collection

Key observational and laboratory indicators were collected during the patients' hospitalization to facilitate comprehensive analysis. Demographic and clinical parameters included gender, age, height, weight, and disease duration. Laboratory data encompassed high-density lipoprotein cholesterol (HDL-C), low-density lipoprotein cholesterol (LDL-C), uric acid, rheumatoid factor (RF), serum C-reactive protein (CRP), erythrocyte sedimentation rate (ESR), and glycated hemoglobin (HbA1c). Additionally, electrocardiographic (ECG) findings were documented, with specific attention to abnormalities such as ST-T changes or the presence of pathological Q waves.

### 2.5 Statistical analysis

Statistical analyses were performed using SPSS software version 27.0 and R software version 4.3.1. Continuous variables following a normal distribution were expressed as mean ± standard deviation ($\bar{x}\pm s$) and compared using the independent *t*-test. Non-normally distributed continuous variables were reported as median (interquartile range) M (Q1, Q3) and analyzed using the Mann–Whitney *U* test. Categorical variables were expressed as frequencies (percentages) and compared using the chi-square (*χ*²) test. Multicollinearity among predictive factors was assessed to eliminate potential direct correlations that could affect the results. Multivariate logistic regression analysis was then employed to identify risk factors associated with CHD in patients with rheumatoid arthritis and to develop a nomogram model. The predictive performance of the model was evaluated using the receiver operating characteristic (ROC) curve, with the area under the curve (AUC) quantifying the model's discriminative ability. Calibration of the model was assessed using the Hosmer-Lemeshow goodness-of-fit test and calibration plots. Decision curve analysis (DCA) was performed to evaluate the clinical utility and net benefit of the model. Internal validation of the model was conducted using the bootstrap resampling method with 1,000 iterations. A *p*-value of <0.05 was considered statistically significant for all tests.

## 3 Results

### 3.1 Comparison of demographic, clinical, and laboratory characteristics between RA + CHD and RA alone groups

The comparison of demographic, clinical, and laboratory characteristics between RA combined with CHD (RA + CHD) and RA alone groups revealed significant differences in several parameters. Patients in the RA + CHD group were significantly older than those in the RA alone group (*P* = 0.002), indicating that age may play a key role in the coexistence of RA and CHD. Additionally, the prevalence of hypertension and ECG abnormalities was markedly higher in the RA + CHD group compared to the RA alone group (*P* = 0.025 and *P* < 0.001, respectively), emphasizing the potential cardiovascular burden associated with RA when complicated by CHD. Regarding metabolic and biochemical parameters, HbA1c levels were significantly elevated in the RA + CHD group (*P* = 0.002), suggesting a stronger association with impaired glucose metabolism in these patients. Similarly, uric acid levels were notably higher in the RA + CHD group (*P* < 0.001), reinforcing its potential role as a cardiovascular risk factor in RA patients. In contrast, there were no significant differences in HDL-C and LDL-C levels between the groups (*P* = 0.514 and *P* = 0.178, respectively) (Table 1). Moreover, smoking status and family history of cardiovascular disease were assessed, with no significant intergroup differences observed (*P* = 0.875 and *P* = 0.902, respectively).

**Table 1:** Comparison of demographic, clinical, and laboratory characteristics between RA + CHD and RA groups.

Parameter	RA combined with CHD (*n* = 32)	RA alone (*n* = 226)	Statistics (t/Z/*χ*²)	*P*-value
Female [*n* (%)]	24 (75.00)	168 (74.34)	0.006	0.936
Smoking Status [*n* (%)]	14 (43.75%)	96 (42.48%)	0.025	0.875
Family History of CVD [*n* (%)]	10 (31.25%)	73 (32.30%)	0.015	0.902
Hypertension [*n* (%)]	19 (59.38)	87 (38.50)	5.049	0.025
ECG Abnormalities [*n* (%)]	20 (62.50)	66 (29.20)	13.98	<0.001
Age ($\bar{x}\pm s$, years)	63.98 ± 6.12	57.65 ± 10.89	3.213	0.002
Body Mass Index ($\bar{x}\pm s$, kg/m²)	23.51 ± 1.98	23.70 ± 2.54	0.406	0.682
RA Duration ($\bar{x}\pm s$, years)	8.94 ± 2.97	7.05 ± 4.10	2.514	0.013
HbA1c ($\bar{x}\pm s$, %)	6.43 ± 1.27	5.92 ± 0.79	3.131	0.002
HDL-C ($\bar{x}\pm s$, mmol/L)	1.26 ± 0.37	1.31 ± 0.41	0.653	0.514
LDL-C ($\bar{x}\pm s$, mmol/L)	2.33 ± 0.60	2.54 ± 0.85	1.350	0.178
Uric Acid ($\bar{x}\pm s$, µmol/L)	312.60 ± 75.89	248.24 ± 90.45	3.837	<0.001
CIMT ($\bar{x}\pm s$, mm)	1.08 ± 0.15	0.94 ± 0.22	3.484	<0.001
Carotid Plaque Crouse Score [M (P25, P75)]	3.02 (1.88, 4.00)	1.53 (0.00, 2.30)	5.658	<0.001
RF [M (P25, P75), IU/ml]	113.00 (25.50, 188.90)	88.00 (28.50, 158.00)	0.867	0.528
CRP [M (P25, P75), mg/L]	19.50 (5.90, 39.85)	14.00 (4.80, 38.00)	0.315	0.213
ESR [M (P25, P75), mm/h]	56.00 (29.50, 101.00)	47.00 (21.00, 74.00)	1.568	0.072

RA, rheumatoid arthritis; CHD, coronary heart disease; HbA1c, glycated hemoglobin; HDL-C, high-density lipoprotein cholesterol; LDL-C, low-density lipoprotein cholesterol; RF, rheumatoid factor; CRP, C-reactive protein; ESR, erythrocyte sedimentation rate; ECG, electrocardiogram; CIMT, carotid intima-media thickness.

Markers of vascular pathology, such as carotid intima-media thickness (CIMT) and carotid plaque Crouse scores, were significantly elevated in the RA + CHD group (*P* < 0.001 for both), indicating more advanced atherosclerotic changes in these patients. These findings highlight the relevance of vascular health monitoring in RA patients at risk for CHD. While the duration of RA was significantly longer in the RA + CHD group (*P* = 0.013), other inflammatory markers such as RF, CRP, and ESR showed no significant differences between the groups (*P* = 0.528, *P* = 0.213, and *P* = 0.072, respectively) (Table 1). This suggests that the presence of CHD in RA patients may be influenced by factors beyond systemic inflammation alone, such as age, metabolic disturbances, and vascular changes.

### 3.2 Multivariate logistic regression analysis of risk factors for CHD in RA patients

The results of the multivariate logistic regression analysis revealed several independent risk factors for CHD in patients with RA. Hypertension emerged as a significant predictor, with an OR of 2.489 (95% CI: 1.048–5.911, *P* = 0.037), highlighting its strong association with CHD in this population. Similarly, elevated HbA1c levels were significantly associated with an increased risk of CHD (OR = 1.624, 95% CI: 1.042–2.529, *P* = 0.033), suggesting a critical role of glucose metabolism in cardiovascular complications. Longer RA disease duration was also independently associated with CHD risk (OR = 1.135, 95% CI: 1.019–1.264, *P* = 0.017), emphasizing the impact of chronic inflammatory processes on cardiovascular health. Atherosclerotic burden, as assessed by the carotid plaque Crouse score, was a significant factor, with an OR of 1.288 (95% CI: 1.041–1.595, *P* = 0.022), indicating that vascular pathology contributes substantially to CHD risk. In terms of biochemical markers, higher uric acid levels were a significant risk factor (OR = 1.005, 95% CI: 1.001–1.010, *P* = 0.014), supporting its potential role as a biomarker for cardiovascular risk in RA patients. Additionally, ECG abnormalities, indicative of underlying cardiac dysfunction, were strongly associated with CHD (OR = 2.553, 95% CI: 1.067–6.110, *P* = 0.033) (Table 2).

**Table 2:** Multivariate logistic regression analysis of risk factors for CHD in RA patients.

Factors	*β* value	Standard error value	Wald value	OR value	95% CI for OR	*P*-value
Hypertension [*n* (%)]	0.912	0.438	4.334	2.489	1.048–5.911	0.037
HbA1c (%)	0.485	0.219	4.930	1.624	1.042–2.529	0.033
RA Duration (years)	0.127	0.053	5.743	1.135	1.019–1.264	0.017
Carotid Plaque Crouse Score (points)	0.253	0.107	5.582	1.288	1.041–1.595	0.022
Uric Acid (*μ*mol/L)	0.005	0.002	6.101	1.005	1.001–1.010	0.014
ECG Abnormalities [*n* (%)]	0.937	0.440	4.543	2.553	1.067–6.110	0.033

RA, rheumatoid arthritis; CHD, coronary heart disease; HbA1c, glycated hemoglobin; ECG, electrocardiogram; OR, odds ratio; CI, confidence interval; SE, standard error.

### 3.3 Development of a nomogram prediction model for CHD in RA patients

**Figure 1:**
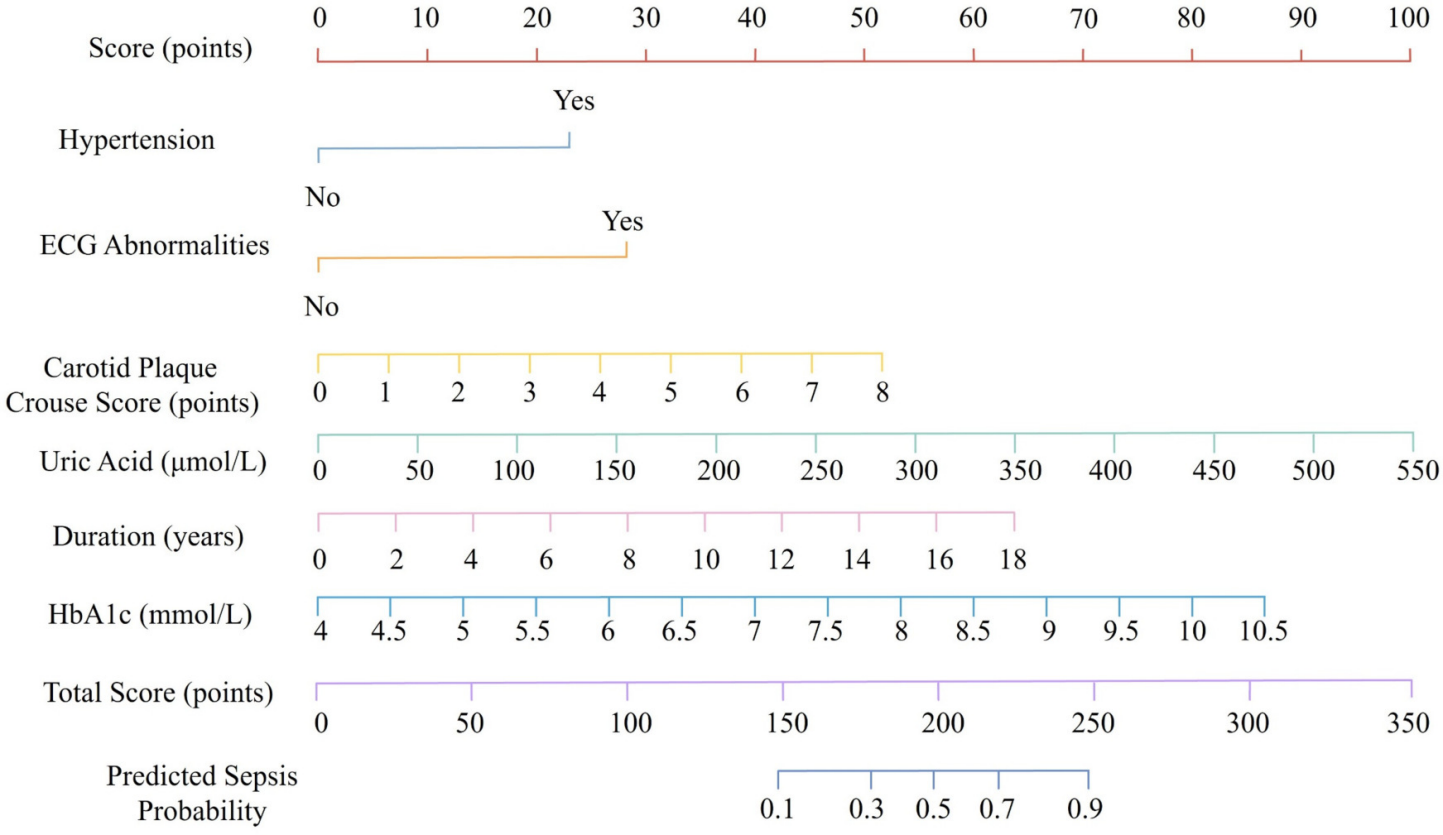
Nomogram for predicting coronary heart disease risk in patients with rheumatoid arthritis.

Based on the results of the multivariate logistic regression analysis, six independent risk factors were identified as significant contributors to the development of CHD in RA patients: hypertension, HbA1c, RA duration, carotid plaque Crouse score, uric acid, and ECG abnormalities. These variables were incorporated into a nomogram prediction model to estimate the probability of CHD occurrence in RA patients. In the nomogram, each predictive variable was assigned a corresponding score based on its relative contribution to CHD risk. The individual scores for all variables were summed to calculate a total score. This total score was then mapped to a probability scale at the bottom of the nomogram, providing an individualized prediction of CHD risk. The higher the total score, the greater the probability of CHD occurrence (Figure 1). This nomogram provides a practical and visual tool for clinicians to assess CHD risk in RA patients by integrating both traditional cardiovascular risk factors and disease-specific variables.

### 3.4 Discriminative ability of the nomogram prediction model for CHD in RA patients

**Figure 2:**
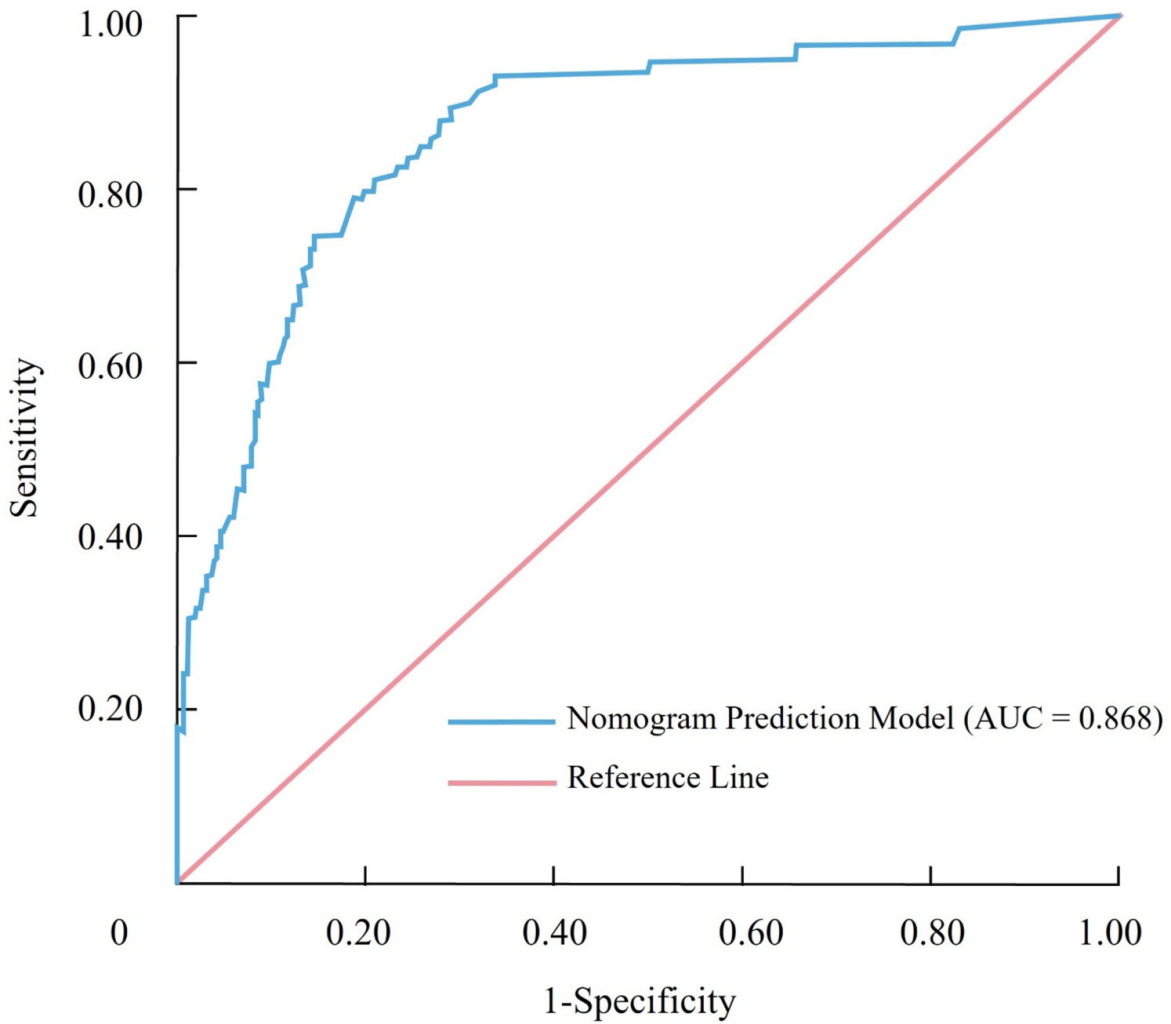
Receiver operating characteristic (ROC) curve illustrating the discriminative performance of the nomogram for predicting coronary heart disease in patients with rheumatoid arthritis.

The discriminative performance of the nomogram prediction model for CHD in RA patients was evaluated using the AUC. The model demonstrated an AUC of 0.868 (95% CI: 0.819–0.916), indicating excellent discriminatory ability. At the optimal cutoff point, determined by the maximum Youden index, the model achieved a sensitivity of 81.2% and a specificity of 83.8%, underscoring its effectiveness in correctly identifying patients at risk for CHD. To ensure the robustness of the model, internal validation was performed using the bootstrap resampling method. Following resampling, the model maintained a high AUC of 0.866 (95% CI: 0.815–0.908), further confirming its reliability and stability. These findings highlight the nomogram's strong discriminative ability, making it a valuable tool for clinical risk stratification of CHD in RA patients (Figure 2).

### 3.5 Calibration of the nomogram prediction model for CHD in RA patients

**Figure 3:**
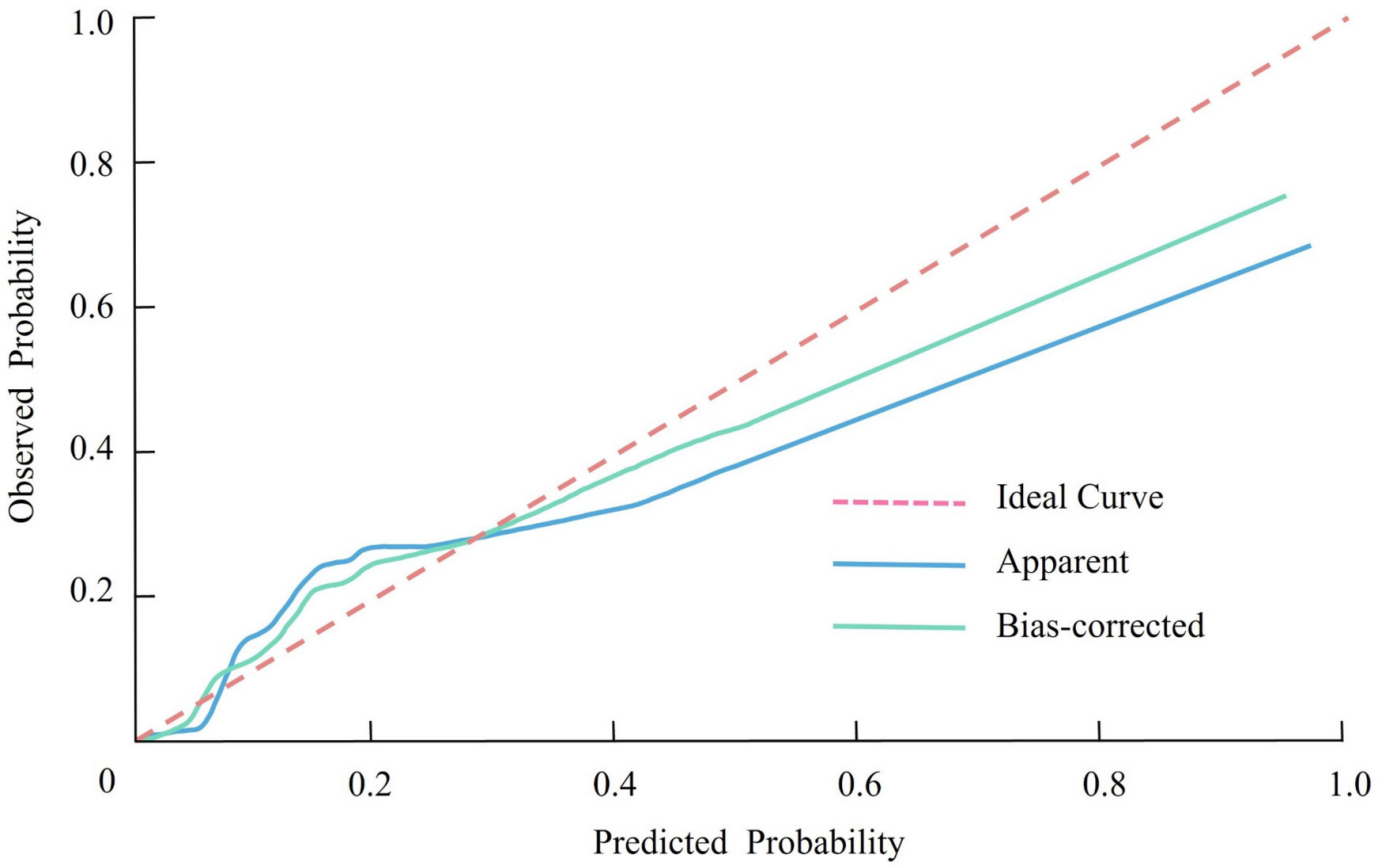
Calibration curve showing the agreement between predicted and observed probabilities of coronary heart disease in patients with rheumatoid arthritis.

The calibration of the nomogram prediction model was assessed using the Hosmer-Lemeshow goodness-of-fit test. The results demonstrated a *χ*² value of 3.188 and a *P*-value of 0.908, indicating that the model's predicted probabilities were well-aligned with the observed outcomes. This suggests that the model has a high degree of calibration and is reliable in estimating the likelihood of CHD occurrence in RA patients. Further confirmation of the model's calibration was provided by the calibration curve (Figure 3). The curve showed that the predicted probabilities closely matched the actual probabilities across different risk levels, demonstrating strong consistency between the predicted and observed outcomes.

### 3.6 Clinical utility of the nomogram prediction model for CHD in RA patients

**Figure 4:**
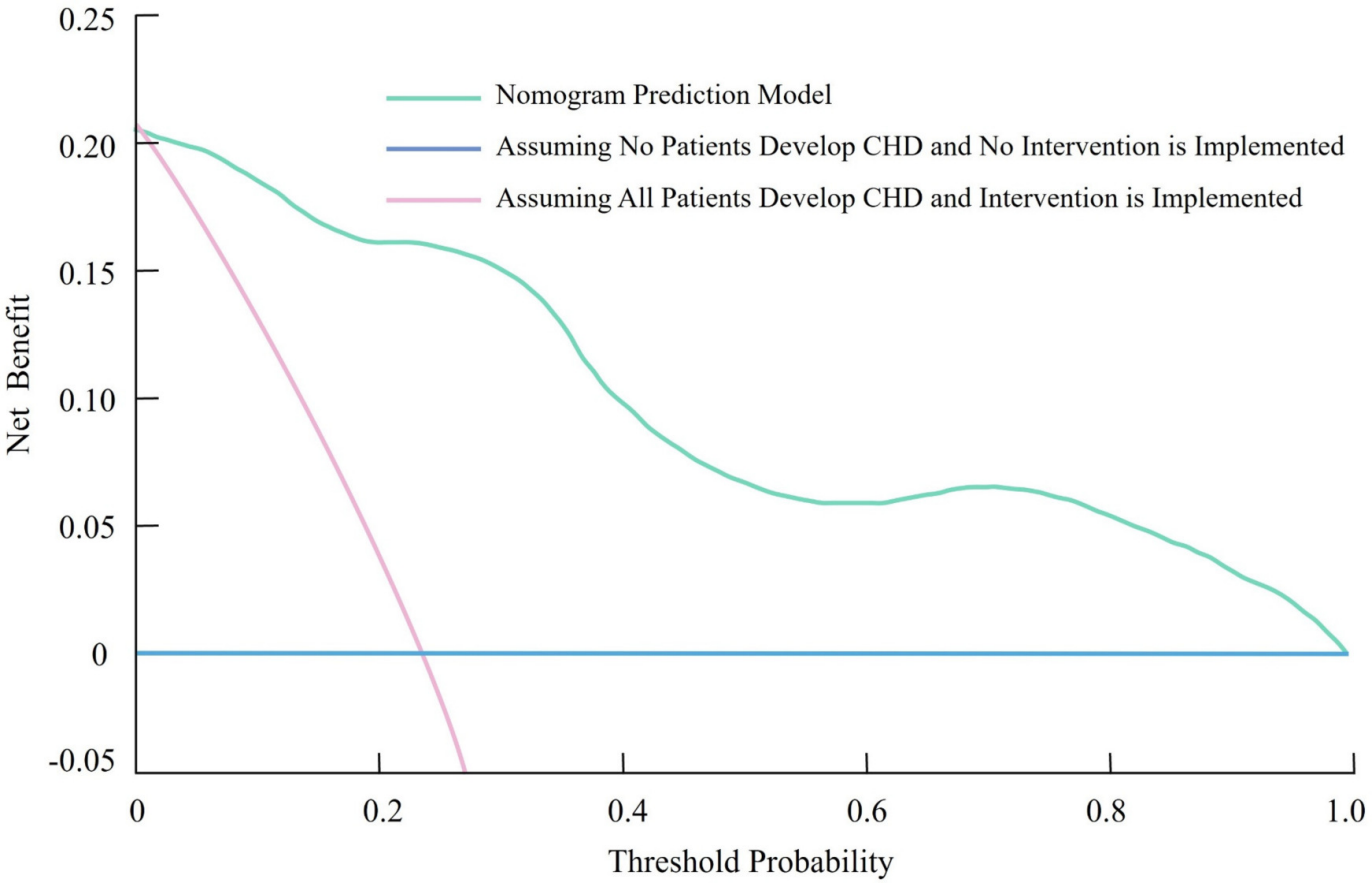
Decision curve analysis (DCA) demonstrating the clinical utility of the nomogram for predicting coronary heart disease in patients with rheumatoid arthritis.

The clinical effectiveness of the nomogram prediction model was evaluated using decision curve analysis (DCA). In the DCA graph, the horizontal line represents the assumption that no patients develop CHD and thus no interventions are implemented, resulting in a net benefit of zero. Conversely, the diagonal line indicates the assumption that all patients develop CHD and are subjected to interventions, leading to a negative net benefit due to unnecessary interventions in patients without CHD (Figure 4). The decision curve demonstrated that the nomogram prediction model yielded a significantly higher net benefit compared to these two extreme scenarios across a wide range of threshold probabilities. This indicates that the model can effectively identify patients at risk for CHD and guide targeted interventions, minimizing unnecessary treatment while optimizing clinical outcomes.

### 3.7 *post-hoc* power analysis

A *post-hoc* power analysis was conducted to evaluate the observed statistical power for the six independent risk factors included in the nomogram model. The analysis was performed based on the effect sizes (Cohen's h for categorical variables and Cohen's d for continuous variables) derived from the group comparisons (RA with CHD vs. RA alone). The individual factors' contribution to the model was weighted according to the normalized coefficients (*β*) obtained from the multivariate logistic regression analysis. The cumulative weighted *post-hoc* power was 0.815, indicating a robust ability to detect significant effects for the risk factors included in the nomogram model. This suggests that the model's performance is statistically reliable and that the sample size was sufficient to identify key predictors of coronary heart disease in rheumatoid arthritis patients.

## 4 Discussion

This study provides a novel contribution to the prediction of CHD in patients with RA by developing a nomogram-based prediction model. The model integrates both traditional cardiovascular risk factors and RA-specific variables, such as disease duration and carotid plaque burden, which have been identified as key determinants of CHD risk in this population (19, 20). The use of a nomogram to predict CHD in RA patients is a significant advancement, as it offers a simple, visual, and individualized tool for risk stratification, allowing clinicians to more accurately identify high-risk patients. The clinical value of this nomogram lies in its ability to quantify the risk of CHD in RA patients, an underserved group at elevated risk for cardiovascular events. By incorporating easily measurable variables such as hypertension, HbA1c, carotid plaque burden, and ECG abnormalities, the nomogram provides a comprehensive risk assessment that is both easy to use and highly applicable to daily clinical practice. This model not only serves to identify patients at risk but also guides clinical decisions regarding surveillance and preventive interventions, such as the initiation of statin therapy or more intensive monitoring for cardiovascular events. The practical utility of this nomogram extends beyond research into direct clinical applications, especially in settings where the prevalence of RA is high. By enabling early identification of patients at high cardiovascular risk, the model can facilitate timely intervention, improving patient outcomes and potentially reducing the burden of CHD in RA patients (21, 22). Furthermore, its straightforward nature makes it an accessible tool for clinicians without requiring specialized software or advanced imaging techniques, which enhances its real-world applicability and scalability.

Several factors were identified as significant predictors of CHD in RA patients, including hypertension, elevated HbA1c, prolonged RA duration, carotid plaque burden, increased uric acid levels, and ECG abnormalities. Hypertension emerged as a critical contributor, consistent with its established role in promoting endothelial dysfunction and atherosclerosis. The chronic inflammatory milieu of RA likely exacerbates hypertension's adverse effects by amplifying vascular stiffness and impairing nitric oxide bioavailability. Impaired glucose metabolism, reflected by elevated HbA1c, was also strongly associated with CHD risk. Persistent hyperglycemia induces oxidative stress, glycation of vascular proteins, and activation of pro-inflammatory pathways, all of which accelerate atherosclerotic plaque formation. The higher HbA1c levels observed in the RA + CHD group may reflect suboptimal glycemic control or heightened systemic inflammation contributing to insulin resistance (23, 24).

The duration of RA was another significant predictor, emphasizing the cumulative impact of chronic inflammation on cardiovascular health. Prolonged exposure to pro-inflammatory cytokines such as tumor necrosis factor-alpha (TNF-α) and interleukin-6 (IL-6) may lead to accelerated vascular damage and plaque instability, increasing CHD risk over time. However, traditional inflammatory markers such as CRP and ESR were not significantly different between groups, suggesting that long-term disease activity, rather than acute inflammation, may play a more pivotal role. IL-6 and TNF-α contribute to cardiovascular risk by promoting endothelial dysfunction, oxidative stress, and vascular remodeling (25). These cytokines play a central role in chronic low-grade inflammation associated with RA and are thought to accelerate atherogenesis and destabilize existing plaques (26). Vascular pathology was evident in the RA + CHD group, with significant elevations in carotid intima-media thickness (CIMT) and carotid plaque Crouse scores (27, 28). These findings underscore the importance of monitoring subclinical atherosclerosis in RA patients. The relationship between uric acid and CHD further highlights the role of metabolic dysregulation, as hyperuricemia promotes endothelial dysfunction and oxidative stress, both of which are implicated in atherogenesis. ECG abnormalities, indicative of myocardial dysfunction or ischemia, were strongly predictive of CHD. This association may reflect the cumulative impact of both systemic inflammation and metabolic disturbances on cardiac health in RA patients.

The nomogram prediction model, incorporating these six independent risk factors, demonstrated excellent discriminative ability with an AUC of 0.868. This suggests the model's potential to accurately stratify CHD risk in RA patients. Internal validation using bootstrap resampling confirmed the model's reliability, with a high AUC of 0.866. The calibration curve further indicated that predicted probabilities closely aligned with observed outcomes, reinforcing the model's accuracy. The nomogram provides a practical tool for personalized risk assessment, enabling clinicians to identify high-risk RA patients who may benefit from intensive cardiovascular monitoring and preventive interventions. DCA demonstrated the model's clinical utility, with significantly higher net benefit across a wide range of threshold probabilities compared to hypothetical extreme scenarios. This highlights the model's ability to optimize decision-making by minimizing unnecessary interventions while ensuring timely management of at-risk patients.

Integrating recent findings with our current results helps to deepen the understanding of inflammation-driven cardiometabolic risk. Barbagallo et al. (29) investigate the impact of LDL receptor (LDLR) mutations on glycemic status and atherosclerosis in familial hypercholesterolemia (FH), finding that LDLR-null individuals have worse glycemic control and more severe coronary artery calcification (CAC). While both studies address cardiovascular risk, Barbagallo et al. focus on genetic mechanisms, highlighting genotype and glycemic status in FH. In contrast, our study identifies traditional cardiovascular risk factors and RA-specific variables, such as disease duration and carotid plaque burden, as key contributors to CHD in RA patients, and presents a nomogram-based predictive model. Similarly, Bosco et al. (30) examine metabolic and immune profiles in FH, showing that individuals with subclinical atherosclerosis (SA) have higher LDL-C levels and dysregulated immune responses. Both studies recognize the interplay between lipid metabolism, immune responses, and atherosclerosis, but our study emphasizes RA-specific factors, such as inflammatory markers, carotid plaque burden, and ECG abnormalities, that contribute to cardiovascular risk in RA patients.

Despite its strengths, this study has several limitations. First, its single-center retrospective design introduces potential selection bias, which may limit the generalizability of the findings. External validation in larger, multicenter cohorts is warranted to confirm the robustness and applicability of the nomogram. Second, while the identified predictors were statistically significant, some—such as hypertension—had relatively wide confidence intervals, reflecting a degree of estimation uncertainty. These associations, though clinically relevant, should be interpreted with caution. Third, although traditional cardiovascular risk factors were included, the role of emerging and RA-specific biomarkers (e.g., homocysteine, adipokines) remains unexplored and should be addressed in future studies. Lastly, the long-term impact of interventions guided by this nomogram on cardiovascular outcomes in RA patients requires further prospective evaluation.

## 5 Conclusions

This study identified key risk factors for CHD in RA patients, including hypertension, HbA1c, RA duration, carotid plaque burden, uric acid, and ECG abnormalities. A nomogram prediction model was developed, demonstrating excellent discriminative and calibration performance. This tool offers a practical approach for individualized CHD risk assessment, facilitating targeted prevention and improving clinical outcomes in RA patients.

## Data Availability

The raw data supporting the conclusions of this article will be made available by the authors, without undue reservation.
